# Effect of NaOH Treatment on the Flexural Modulus of Hemp Core Reinforced Composites and on the Intrinsic Flexural Moduli of the Fibers

**DOI:** 10.3390/polym12061428

**Published:** 2020-06-26

**Authors:** Ferran Serra-Parareda, Francesc Xavier Espinach, Maria Àngels Pelach, José Alberto Méndez, Fabiola Vilaseca, Quim Tarrés

**Affiliations:** 1 LEPAMAP Research Group, University of Girona, Maria Aurèlia Capmany, 61, 17003 Girona, Spain; angels.pelach@udg.edu (M.À.P.); joaquimagusti.tarres@udg.edu (Q.T.); 2 Design, Development and Product Innovation, Department of Organization, Business, University of Girona, Maria Aurèlia Capmany, 61, 17003 Girona, Spain; francisco.espinach@udg.edu; 3 Advanced Biomaterials and Nanotechnology, Department of Chemical Engineering, University of Girona, Maria Aurèlia Capmany, 61, 17003 Girona, Spain; fabiola.vilaseca@udg.edu; 4 Department of Industrial and Materials Science, Chalmers University of Technology, SE 412 96 Gothenburg, Sweden; 5 Sustainable Industrial Processes, University of Girona, Maria Aurèlia Capmany, 61, 17003 Girona, Spain

**Keywords:** hemp core, intrinsic flexural modulus, composites, chemical treatment, polypropylene

## Abstract

This paper describes the potential of using hemp core waste in the composite industry. These lignocellulosic residues can be used to produce environmentally friendly and economically viable composites and improve the overall value chain of hemp production. To this purpose, hemp core residues were alkaline treated at different NaOH concentrations and then mechanically defibrated. Hemp core fibers were mixed with polypropylene and injection molded to obtain testing specimens. The effect of sodium hydroxide on the flexural modulus of composites was studied from macro and micro mechanical viewpoints. Results showed remarkable improvements in the flexural modulus due to the presence of hemp core fibers in the composites. At a 50 wt % of reinforcement content, increments around 239%, 250% and 257% were obtained for composites containing fibers treated at a 5, 7.5 and 10 wt % of NaOH, respectively. These results were comparable to those of wood composites, displaying the potential of hemp core residues. The intrinsic flexural modulus of the hemp core fibers was computed by means of micromechanical analysis and was calculated using the ratios between a fiber flexural modulus factor and a fiber tensile modulus factor. The results agreed with those obtained by using models such as Hirsch and Tsai–Pagano. Other micromechanical parameters were studied to fully understand the contribution of the phases. The relationship between the fibers’ intrinsic flexural and Young’s moduli was studied, and the differences between properties were attributed to stress distribution and materials’ anisotropy.

## 1. Introduction

Natural fiber composites have become an important topic for the scientific community and industry. Natural fibers offer several benefits over conventional mineral reinforcements, such as low production costs, low energy consumption requirements, low specific weight, environmental friendliness and global contribution to achieve sustainable development. Adversely, synthetic reinforcements like glass fibers entail health risks and promote abrasion to manufacturing equipment [1,2,3,4,5].

Within the field of composite materials, the demand for agroforestry residues as reinforcement has increased recently. This is mainly due to social environmental awareness related to the management of wood resources, to avoid deforestation and its inherent impact on biodiversity. Additionally, reusing lignocellulosic residues can prevent stubble burning and avoid CO_2_ emissions. In this context, the European Union has proposed great challenges in relation to waste management strategies, one of the major goals in the transition towards a circular economy. This approach is based on a “waste hierarchy” system, defining different options in the following priority order: Prevention, reuse, recycling, recovery, disposal (landfilling or incineration) [6,7,8]. Accordingly, it is important to devise different applications for lignocellulosic residues, and at the same time consider the economic and environmental impact of its management. Therefore, the valorization of these residues in added value applications such as the composite industry is an important target in materials research [9,10,11,12].

**Figure 1:**
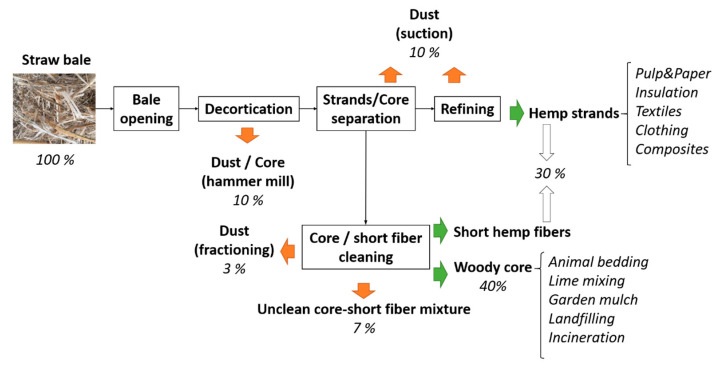
Industrial processing of hemp straw [14,15].

Industrial hemp core residues are particularly attractive due to their vast availability and low cost, retailing for less than 0.2$/kg [13]. Hemp core residues are obtained during the production of hemp strands. While the large content of cellulose and elevated aspect ratios of the strands allows its use in a variety of valuable applications, from the papermaking to the textile industries, the hemp core remains a residue. The industrial processing of hemp straw is presented in Figure 1.

Decortication processes aim at removing the outer fibrous layers from the inner core layers (Figure 1). Hammer mills used for hemp decortication demand high amounts of energy, making the whole process costly. Hence, the valorization of hemp core residues in added value applications can make hemp processing more economically profitable. After decorticating, the strands are separated from the mixture, leaving a mix of short fibers and core, which is further classified. Hemp core represents a 40 wt % of the initial biomass, whereas the combination of strands and short fibers account for a 30 wt %. The rest is dust produced during the decortication process. As a result, huge quantities of hemp core remain as residue. In fact, according to the European Industrial Hemp Association (EIHA), in Europe, where 85% of the world hemp cultivation is located, about 85,000 tons of hemp are harvested and manufactured annually, resulting on average in 43,000 tons/year of hemp core.

Hemp core residues are principally devoted to low value applications, such as animal bedding, mainly because of the high capacity to adsorb liquids. However, hemp core residues have recently drawn the attention of researchers as a potential source of reinforcements for polymer-based composites and a valuable substitute for wood in wood–plastic composites [14,15,16,17]. The main drawbacks of hemp core fibers compared to wood fibers are an elevated content of lignin (21–24 wt %) along with low aspect ratios, which can lead to composites with weak interfacial adhesion. If there is weak adhesion across the boundary phase, the stress is inefficiently transferred through the reinforcement, resulting in composites with poor mechanical properties. Hence, the interfacial adhesion issue should be solved if the purpose is to open new market opportunities to valorize the residue.

Obtaining a strong interfacial bonding has generally been pursued considering two approaches: Fiber treatments [18,19] and the use of compatibilizers [20,21]. Alkaline treatments involving sodium hydroxide are the most widely used due to the reasonable affordability of the reagents and their effectiveness. NaOH treatments aim at the removal of fiber constituents such as lignin, extractives, some hemicelluloses and inorganic matter, increasing the (crystalline) cellulose content [22,23,24,25]. Soft NaOH concentrations from 0 to 10 wt % have proved to increase the fiber crystallinity by removing amorphous substances and incrementing the cellulose content. On the contrary, harsher treatments over the 10 wt % of NaOH can result in excessive fiber delignification, finally damaging or weakening the fiber cell wall and driving to chain breakage and final reduction of fibers’ intrinsic properties [26,27,28]. Regarding process yields, soft NaOH treatments involve lower waste generation, which is a goal of the circular economy and agrees with the principles of green chemistry [29]. The fiber’s morphology is also affected by lignin removal, since fiber individualization is promoted, achieving higher aspect ratios and contributing to the enhancement of the mechanical properties [30,31].

On the other hand, the reduction of extractives and lignin increases the presence of hydroxyl groups on the fiber surface. By using maleic anhydride polymers as compatibilizers it is possible to generate chemical bonds between the phases to improve the stress-transfer efficiency in composites. These compatibilizers link hydroxyl groups exposed in the fiber surface by means of maleic groups. On the other hand, the compatibilizer interacts with the unmodified polymer chains via a self-entangling mechanism. The literature about natural fiber reinforced polypropylene composites shows that the use of maleic anhydride polypropylene (MAPP) at the proper percentages strengthens the interfacial adhesion [32,33,34].

When envisioning the application of newly developed materials, it is of relevance that such materials meet the industrial demands required for a certain application. In this sense, sectors such as automotive and construction are two examples where natural fiber-based composites are currently used. Products such as door panels, seat backs, headliners, dashboards, roofing sheets, windows and tiles, among others, are being commercialized. In these cases, stiffness is more important than the ultimate strength of the materials. In fact, according to the literature, both stiffness and dimensional stability are the most influential parameters determining the industrial viability of composite materials [10,35]. Besides, products typically addressing building and structural/semi-structural applications are usually subjected to flexural loads, whereas purely tensile cases are scarce in comparison. This makes designers particularly interested in previewing the materials’ behavior under flexural loads [36,37]. Therefore, knowing the flexural properties of the materials, and specifically its flexural moduli, is of interest to know the competitiveness of a material.

The present paper explores the potential competitiveness of hemp core residues as reinforcement to polypropylene-based composites. With this purpose, hemp core residues were treated under different NaOH concentrations and later defibrated using Sprout–Waldron equipment. The obtained fibers were mixed with polypropylene at different contents, ranging from 10 to 50 wt %. Polypropylene is one of the most widely used polymers for the preparation of composites. Polypropylene’s main advantages are its strength and stiffness, weather and chemical resistance, good corrosion, low specific weight and low cost [38]. MAPP was added to the composites formulation to enhance the fiber–matrix interfacial adhesion. The effect of the NaOH treatments on the flexural moduli of the composites was tested and discussed. In addition, a micromechanical analysis was projected to find out the intrinsic flexural modulus of the fibers, among other relevant micromechanical factors. To the best of our knowledge, the flexural modulus of hemp core fiber-reinforced composites is yet to be explored, and the intrinsic flexural modulus of fibers is unknown in the literature.

## 2. Materials and Methods

### 2.1. Materials

Composite materials were prepared using hemp core fibers as reinforcement and polypropylene as the polymer matrix. Polypropylene PP ISPLEN 090 GDM (PP) was purchased from Repsol S.A (Tarragona, Spain). Hemp core residues were kindly supplied by Agrofibra S.L. (Puigreig, Spain). The coupling agent used in the present work was a maleic anhydride polypropylene (MAPP) under the trade name Epolene G 3015 (Eastman Chemical Products, San Roque, Spain).

Sodium hydroxide (NaOH) and anthraquinone (AQ) were employed for the chemical treatment, both supplied by BASF (Tarragona, Spain). The recovery of the fiber from the composite material was carried out using decahydronaphthalene (decalin) to dissolve polypropylene. Decalin, was provided by Sharlab S.L. (Sentmenat, Spain). Reagents were used as received.

### 2.2. Methods

#### 2.2.1. Hemp Core Fibers Treatment

Hemp core residues were subjected to chemo-thermomechanical treatment, which involved fibers’ digestion by means of NaOH and AQ followed by mechanical defibration using Sprout–Waldron equipment.

Three different digestions containing 5, 7.5 and 10 wt % of NaOH, with respect to fiber content, were prepared to evaluate how the intensity of the treatment affected the stiffening abilities of the fibers. AQ was added as catalyzer at 0.1 wt % concentration to enhance the chemical degradation of lignin. The digestions were carried out at 10:1 liquid to solid ratios, a temperature of 98 ± 2 °C, 90 min and 15 L batch volume. The resulting fibrous suspensions were profusely washed with deionized water to remove impurities and then passed through a Sprout–Waldron mechanical defibrator to individualize the fibers. Finally, the suspensions were filtered and oven-dried at 80 °C until constant weight.

#### 2.2.2. Composite Preparation and Sample Obtaining

Composites with reinforcement amounts ranging from 10 to 50 wt % were prepared by means of a Gelimat kinetic mixer model G5S (Draiswerke, NJ, USA). Gelimat is high-speed melt mixing equipment which achieves the blending of the materials in few minutes (1–2 min). The mixture is rapidly discharged when the melting temperature of the polymer is reached, since the shearing forces increase abruptly. Thereby, fibers are exposed for a few seconds to elevated temperatures, minimizing possible side effects. In addition, the mixer promotes the dispersion and distribution of the fibers, reducing fiber attrition in comparison to other internal mixers.

Initially, the oven-dried fibers were added at 300 rpm. Then, polypropylene and MAPP were incorporated, maintaining this constant speed. Afterwards, the speed was increased up to 2500 rpm until the phases were completely melted and mixed. The process lasted about 2 min, after which time a temperature of 195 °C was achieved. The mixture was discharged and left outside to cool down.

Next, the material was pelletized in a knives mill fitted with a 5 mm mesh. The material was kept at 80 °C prior to further processing to prevent moisture uptake. Specimens for the flexural test were procured using a steel mold in an injection molding machine Aurburg 220 M 350-90U (Aurburg, Loßburg, Germany), complying with ASTM D3641 specifications. The injection molding process was carried out at 180, 185, 190, 195 and 195 °C, the last being the injection nozzle. First and second pressures were 120 and 37.5 kg·cm^−2^, respectively. Higher temperatures were avoided to avoid fiber degradation. When fiber content was increased in the composites, little increases in first pressure were necessary for the correct injection of specimens.

**Figure 2:**
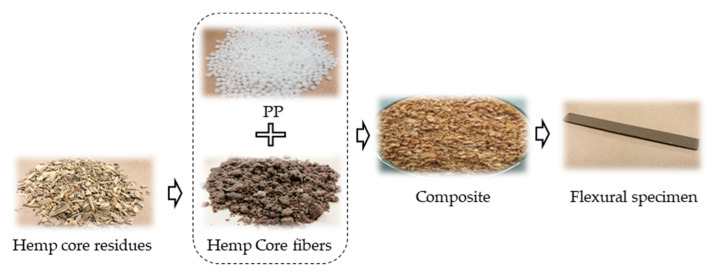
Illustrations of the raw materials (polypropylene PP ISPLEN 090 GDM (PP), hemp core), hemp core fibers after being treated, composite material and flexural specimen 2.2.3. Three-point bending test.

For the readers’ convenience, images of the raw materials (PP, hemp core), hemp core fibers after being treated and composite materials are shown in Figure 2.

Before testing, specimens were stored in a conditioning chamber (Dycometal) at 23 °C and 50% relative humidity for 48 h, as required by ASTM D618. Then, specimens were subjected to three-point bending test by means of an Instron universal testing machine equipped with a 5 kN load cell, in accordance with ASTM D790. The flexural deformation and flexural modulus at each composite formulation were measured from the average of at least five sample tests.

#### 2.2.3. Characterization of the Fibers

The degrees of delignification of hemp core fibers at the different NaOH concentrations were assessed via the Kappa number according to TAPPI T 236 om-06. Kappa number is a rapid and simple method used to evaluate the lignin content of the pulp.

The reinforcing fibers were recovered from the composite materials by matrix dissolution in decalin using a Soxhlet apparatus. The fiber extraction was performed for 48 h. Then, fibers were rinsed with acetone and deionized water to remove the impurities and residual decalin. Fibers were then suspended in water to avoid aggregation. Morphological data of the fibers was procured by means of a MorFi Compact analyzer (Techpap, Grenoble, France). The analysis provided the mean fiber length and diameter. The equipment allowed the acquisition of 30,000 images per test, recording a total of four tests for each type of fiber. Light microscopy images were recorded by means of optical microscope (Leica, model DMR-XA, Barcelona, Spain) in order to obtain a qualitative analysis of the morphological composition of the fibers. Scanning electron microscopy (SEM) (Zeiss, DSM 960, Madrid, Spain) was performed at the cross-sectional area of the fracture surface of flexural specimens.

The composites’ density was determined using a pycnometer following ISO 1183–1. The fibers’ density was calculated from the following relationship, $\rho^{c}=w^{c}/\left(w^{m}/\rho^{m}+w^{F}/\rho^{F}\right)$, where $w^{c}$, $w^{m}$ and $w^{F}$ are, respectively, the contents in weight of the composite, matrix and fiber. Moreover, $\rho^{F}$ and $\rho^{m}$ are the fibers and matrix densities. From the density measurements, the fiber volume fraction at each fiber loading was $V^{F}=(w^{F}/\rho^{F})/\left(w^{m}/\rho^{m}+w^{F}/\rho^{F}\right)$.

### 2.3. Modeling the Flexural Modulus

Despite all existing information and knowledge about natural fiber composites, their complex behavior under load, different to traditional materials, and their anisotropy require an in-depth study to improve their performance and take advantage of their potential. To this purpose, micromechanical models provide information on the expected properties of composite materials as the sum of the contributions of their phases.

A modified Rule of Mixtures (mRoM) is one of the simplest and most used methods to compute the contributions of the phases to a mechanical property of a composite. The model was initially established for the prediction of the Young’s modulus [39], though it was further adapted to the flexural modulus [40,41]. An mRoM for the flexural modulus is shown in Equation (1).

(1) $$E_{f}^{c}=\eta_{e}\cdot E_{f}^{F}\cdot V^{F}+E_{f}^{m}\cdot \left(1-V^{F}\right)$$

where $E_{f}^{c}$, $E_{f}^{F}$ and $E_{f}^{m}$ are the flexural modulus of the composite, fiber and matrix, respectively. The modulus efficiency factor $\eta_{e}$ is incorporated into the equation to correct the contribution of fibers to the flexural modulus of composites due to their mean orientation and morphology.

At its current shape, the mRoM (Equation (1)) contain two unknowns, which are the modulus efficiency factor $\left(\eta_{e}\right)$ and the intrinsic flexural modulus $\left(E_{f}^{F}\right)$. The intrinsic flexural modulus of the reinforcing fibers was calculated via two different methodologies: (i) Hirsch model and (ii) Tsai–Pagano model using Halpin–Tsai equations, namely TP&HT.

The Hirsch model combines the parallel model developed by Reuss, and the series model by Voigt [42]. The parallel model describes a system where the load occurs longitudinally in the fiber direction, whereas in the serials models the load is perpendicular to the fiber. Both models are brought together by the inclusion of a stress-transfer coefficient (β), which mainly depends on the fiber length, fiber orientation and fiber distribution inside the matrix. Values close to β = 0.4 have shown good agreement between theoretical and experimental values [25]. The Hirsch model is described as follows:

(2) $$E_{t}^{c}=\beta \cdot \left(E_{f}^{F}\cdot V^{F}+E_{f}^{m}\left(1-V^{F}\right)\right)+\left(1-\beta \right)\cdot \;\frac{E_{f}^{F}\cdot E_{f}^{m}}{E_{f}^{m}\cdot V^{F}+E_{f}^{F}\cdot \left(1-V^{F}\right)}$$

Unlike the Hirsch model, the Tsai–Pagano model [43] includes morphologic data of the fibers by the incorporation of Halpin–Tsai equations [44]. The Tsai–Pagano model is represented by:

(3) $$E_{t}^{c}=\frac{3}{8}E^{11}+\frac{5}{8}E^{22}$$

where $E^{11}$ and $E^{22}$ are the flexural modulus in the longitudinal and transverse direction of the loads. Both moduli can be obtained from Halpin–Tsai equations.

(4) $$E^{11}=\frac{1+2\left(l_{ww}^{F}/d^{F}\right)\cdot \eta_{l}V^{F}}{1-\eta_{l}V^{F}}\cdot E_{f}^{m}\;with\;\eta_{l}=\frac{\left(E_{f}^{F}/E_{f}^{m}\right)-1}{\left(E_{f}^{F}/E_{f}^{m}\right)+2\left(l_{ww}^{F}/d^{F}\right)}$$

(5) $$E^{22}=\frac{1+2\eta_{t}V^{F}}{1-\eta_{t}V^{F}}\cdot E_{f}^{m}\;with\;\eta_{t}=\frac{\left(E_{f}^{F}/E_{f}^{m}\right)-1}{\left(E_{f}^{F}/E_{f}^{m}\right)+2}$$

In the equations, $l_{ww}^{F}$ and $d^{F}$ are the mean doubly weighted fiber length and mean fiber diameter. Similarities between Hirsch and Tsai–Pagano and Halpin–Tsai models were studied by means of ANOVA statistical model. Knowing the intrinsic flexural modulus, it is possible to find out the modulus efficiency factor from the mRoM (Equation (1)). The efficiency factor is mainly influenced by the morphology and mean orientation of the fibers. In fact, the term is decomposed into two factors, a length efficiency factor $\left(\eta_{l}\right)$ and an orientation efficiency factor $\left(\eta_{o}\right)$.

(6) $$\eta_{e}=\eta_{l}\cdot \eta_{o}$$

In the case of mold-injected short fiber semi-aligned composites, the orientation factor delivers values between 0.4 and 0.6 and is mostly affected by the compounding conditions and mold geometry in the injection machine [45]. Besides, the length factor relates to fiber morphology [34]. The length factor $\left(\eta_{l}\right)$ can be calculated from the expressions developed by Cox [46] and Krenchel [47], and further reviewed by numerous authors [48,49,50].

(7) $$\eta_{l}=1-\frac{\tan h\left(\xi \cdot l_{ww}^{F}/2\right)}{\xi \cdot l_{ww}^{F}/2}$$

where ξ is the stress concentration rate at the ends of fibers, defined as:

(8) $$\xi =\frac{1}{\left(\frac{d^{F}}{2}\right)}\sqrt{\frac{E_{f}^{m}}{E_{f}^{F}\cdot \left(1-\nu \right)\cdot \ln{\left(\frac{\pi }{4\cdot V^{F}}\right)}^{\frac{1}{2}}}}$$

where ν is the Poisson’s coefficient of PP, with a value of 0.36 [51,52]. Once the efficiency and length factors are computed, the orientation factor can be obtained from Equation (6).

Fukuda and Kawata [53] proposed a methodology linking the mean orientation factor and a defined limit angle of the fibers $\left(\alpha_{o}\right)$ inside the matrix, assuming square packing distribution of the reinforcement. The relation is the following.

(9) $$\eta_{o}=\frac{\sin\left(\alpha_{o}\right)}{\alpha_{o}}\left(\frac{3-\upsilon }{4}\cdot \frac{\sin\left(\alpha_{o}\right)}{\alpha_{o}}+\frac{1-\upsilon }{4}\cdot \frac{\sin\left(\alpha_{o}\right)}{\alpha_{o}}\right)$$

Besides, Sanomura and Kawamura [48] established a connection between the defined limit angle $\left(\alpha_{o}\right)$ and the theoretical mean orientation angle of fibers (α) by means of an orientation factor $\left(f_{p}\right)$, as presented in Equation (12).

(10) $$f_{p}=\frac{\sin\left(2\alpha_{o}\right)}{2\alpha_{o}}=2\cdot \cos^{2}\left(\alpha \right)-1$$

**Figure 3:**
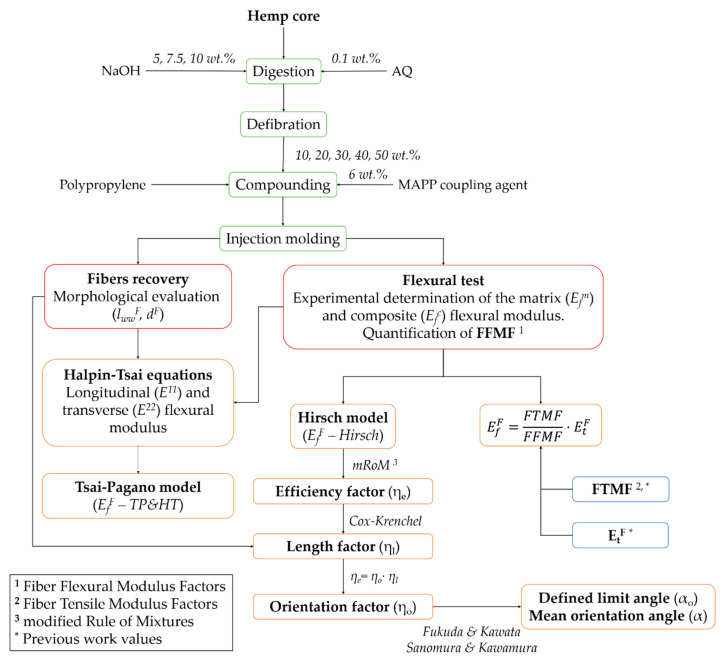
Investigation work plan.

Towards a better understanding, Figure 3 schematically presents the work plan followed in the present investigation, from the manufacturing of the materials to the final macro and micro mechanical characterization of the flexural modulus.

## 3. Results

### 3.1. Effect of Sodium Hydroxyde (NaOH) Treatment on Hemp Core Fibers

The purpose of the soft sodium hydroxide (NaOH) treatment catalyzed with anthraquinone (AQ) was the removal of lignin, most extractives, ashes and some hemicelluloses from the fiber surface. The catalyzer (AQ) enhances the degradation of lignin to promote its release. The effect of NaOH concentration (5, 7.5 and 10 wt %) on the lignin content and morphology of the fibers was evaluated. Such factors are known to affect the performance of natural fibers as composite reinforcements (Table 1). The raw biomass, formed by large amounts of fiber bundles, was impossible to properly individualize. Hence, its initial characterization was meaningless.

The Kappa number decreased as the NaOH concentration increased. Hence, the alkaline treatments offered an effective lignin removal. More precisely, the Kappa number of the 10 wt % NaOH-treated fibers was 5% and 16% lower than the ones treated at a 7.5 and 5 wt % of NaOH, respectively. Besides, the manufacturing yield, identified as the weight ratio between the raw biomass and the resulting fibers, also decreased with the intensity of the treatment. The yield rendered values close to 70%. According to the principles of green chemistry and EU waste management legislation, residue generation must be limited and further reused or recycled if possible [29]. In the present case, the yields obtained were in the lower range of high-yield lignocellulosic processing methods, ensuring the minimum generation of residues [11]. Thereby, higher concentrations of NaOH were discarded as lower yields were expected. Recent works show the potential of lignin resulting from fibers’ digestion as a source of carbon fibers [54,55,56] or can be used for other purposes such as concrete additive, thermoplastic material and production of fuels, among others [57,58]. These uses show the possibilities of minimizing the overall generation of residues and close the ecological and economical loop.

**Figure 4:**
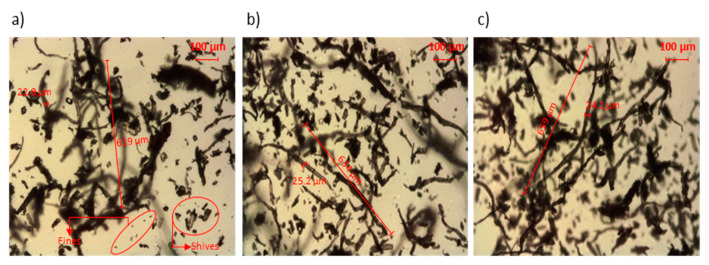
Light microscopy images of hemp core fibers treated at (**a**): 5 wt % NaOH, (**b**): 7.5 wt % NaOH and (**c**): 10 wt % NaOH.

The morphology of the fibers did not change noticeably with the amount of NaOH. Only slight increments in the mean fiber length were attained. The differences in fiber length were statistically significant as determined by ANOVA analysis at 95% confidence rate. Besides, the ANOVA analysis revealed no significant differences between fibers’ diameters. The morphological analysis agrees with the observation in the light microscopy images (Figure 4). In addition, the light microscopy images indicated the presence of short fibers, fines and shives. Overall, the aspect ratios were superior to 10 in all cases, suggesting the strengthening and stiffening potential of the fibers [59,60].

### 3.2. Macro-Mechanics of the Flexural Modulus

Composites’ flexural modulus depends on the fiber and matrix properties, reinforcement content, fiber mean orientation and the grade of dispersion of the fibers inside the matrix [41]. The strength at the fiber–matrix interphase has proved to have little effect on the flexural modulus of composites for various thermoplastics [10,50,61,62]. Nonetheless, the inclusion of coupling agents to promote the interfacial bonding is largely recommended to enhance the material’s strength and deformation ability [32,49,63]. Previous works analyzing the tensile properties of hemp core fiber-reinforced polypropylene composites proposed the amount of MAPP required to enhance the interfacial adhesion at 6 wt % with respect to fiber content [64,65]. The experimental values of the flexural modulus and flexural deformation at break are presented in Table 2.

The presence of the coupling agent in the formulation showed little effect on the flexural modulus, corroborating the strength of the interphase is not critical for the flexural modulus. However, coupled composites were able to withstand higher deformations in comparison to uncoupled ones. As a result, the addition of MAPP to the formulation is viewed as an industrial competitive advantage over uncoupled composites.

Composites containing fibers treated at 10 wt % of NaOH delivered slightly higher flexural modulus values than the ones obtained at lower, 5 and 7.5 wt %, NaOH concentrations. In fact, a remarkable increment of the flexural modulus about the 257% was attained for composites at 50 wt % of fiber content. Comparatively, Shah et al. investigated the flexural modulus of hemp strand-reinforced polypropylene and an increment by 10–15% was recorded after a 30 wt % fiber addition [66]. The low increments were ascribed to strong heterogeneity in the microstructure, in terms of fiber orientation. Besides, the NaOH effect agrees with recent literature dealing with other plant fibers. For example, a favorable concentration of 20 wt % NaOH was reported for coir fiber-reinforced polyester composites. Sisal fiber was also used as reinforcement for polypropylene, attaining the best mechanical properties at a 10 wt % of soda [67].

**Figure 5:**
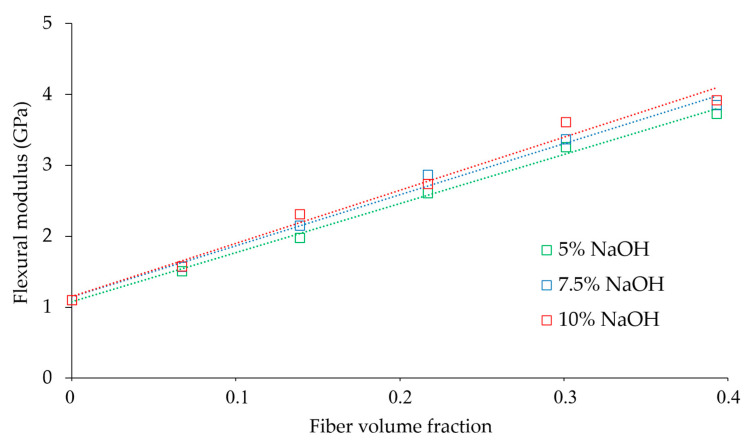
Evolution of the flexural modulus with the fiber volume fraction.

The flexural modulus evolved linearly with the fiber volume fraction in composites containing hemp core fibers treated at 5 and 7.5 wt % of NaOH. The flexural modulus of composites charged up with fibers treated at 10 wt % of NaOH increased linearly up to 40 wt % of reinforcement, then the flexural modulus of the composite at 50 wt % moves away from such linear behavior (Figure 5). This is explained by the tendency of more severely delignified fibers to self-aggregate, forming fiber bundles at elevated fiber contents. Fibers’ agglomeration influences the impregnation of the polymer on the fibers, resulting in lower dispersion of the reinforcement inside the matrix. Subsequently, the stress-transfer between the phases drops, hindering the increment of mechanical properties. This problem is particularly prone to composites using polyolefin as matrices, since its high hydrophobicity contrasts significantly with the hydrophilicity of natural fibers [68].

**Figure 6:**
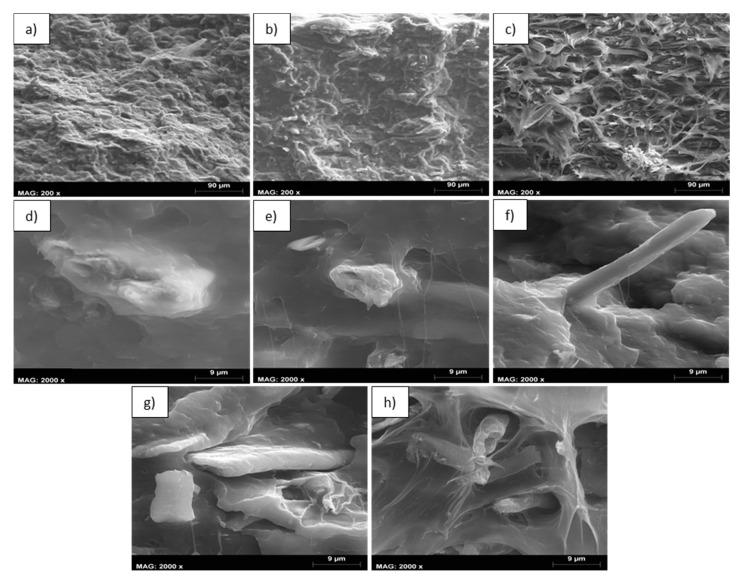
Scanning electron microscopy images of the cross-sectional area of the flexural specimens. (**a**) PP+20% hemp—5% NaOH (200×), (**b**) PP+20% hemp—7.5% NaOH (200×), (**c**) PP+20% hemp—10% NaOH (200×), (**d**) PP+20% hemp—5% NaOH (2000×), (**e**) PP+20% hemp—7.5% NaOH (2000×), (**f**) PP+20% hemp—10% NaOH (2000×), (**g**) PP+50% hemp—5% NaOH (2000×) and (**h**) PP+50% hemp—10% NaOH (2000×).

Overall, favorable lignin amounts, being the most hydrophobic compound in the fiber with most affinity with the hydrophobic polymer, can help the dispersion and distribution of the fibers inside the reinforcement. Certainly, the most linear correlation (R^2^ = 0.9961) was attained in composites with fibers with the highest Kappa number (5 wt % NaOH), where the dispersion of the reinforcement was supposedly superior. This is viewed as a competitive advantage compared to other natural fibers, since the possibility of adding larger amounts of such natural fibers saves part of the polymer. Hence, the mechanical property does not decline, and a reduction of the material’s cost can be achieved. To further understand how the concentration of NaOH and fiber content influence the flexural modulus, SEM images were added showing the morphology and distribution of the fibers inside the composite. The images were taken at the cross-sectional area of the specimens after being fractured during the three-point bending test. The images are shown in Figure 6.

As the severity of the NaOH treatment was increased, the cross-sectional area of the flexural specimens became more irregular (Figure 6c) and more voids could be observed. In contrast, specimens charged with fibers treated at lower NaOH concentrations (Figure 6a,b) showed a smoother and more well-preserved structure. As mentioned above, this is attributed to a better dispersion and distribution of the fibers treated at lower NaOH concentrations, since the lignin content remains higher and it helps the dispersion inside the matrix. At higher magnifications (2000×), it is possible to see that those fibers treated at 5% NaOH (Figure 6d) adhere more to the matrix. In fact, it was quite difficult to locate the fibers in this sample during SEM observation because of its high dispersion. Besides, at severer NaOH treatments the dispersion and adhesion to the matrix decreased progressively, as reflected in Figure 6e,f.

Images at higher fiber loading (50 wt %) and magnification (2000×) were also acquired. Differences were detected between fibers treated at the same NaOH concentration of 5% and different fiber loading (20 and 50 wt %) inside the composite (Figure 6d,g). The increase of reinforcement content led to lower dispersion and distribution of the fibers inside the matrix. A similar behavior was observed at higher NaOH concentrations (10% NaOH) and fiber contents of 20 and 50 wt % (Figure 6f,h). Between composites charged with elevate fiber contents (50 wt %) and different NaOH concentrations of 5 and 10% (Figure 6g,h), it is observed that those fibers treated at higher NaOH amounts exhibited lower dispersion and adhesion with the matrix.

### 3.3. Contribution of the Fibers to the Flexural Modulus

When a composite material is developed, there is an interest in obtaining a parameter that allows determining the contribution of the fibers to the mechanical properties of the material. Such a parameter can be obtained from an mRoM (Equation (1)). In the equation, the net contribution of the reinforcement is expressed by $\eta_{e}\cdot E_{f}^{F}\cdot V^{F}$, whereas the contribution of the matrix is computed by $E_{f}^{m}\cdot \left(1-V^{F}\right)$. In this regard, Thomason [69] established a methodology to quantify the net contribution of the fibers to the flexural modulus, which would be independent from the type of matrix used and fiber loading, thus, serving for the comparison with other plastic-based composites. By plotting the difference between the composite’s flexural modulus and the contribution of the matrix, expressed as $E_{f}^{c}-E_{f}^{m}\cdot \left(1-V^{F}\right)$, with the fiber volume fraction $\left(V^{F}\right)$ in each of the composites, a Fiber Flexural Modulus Factor (FFMF) is obtained from the slope of the line.

(11) $$FFMF=\eta_{e}\cdot E_{f}^{F}=\frac{E_{f}^{c}-E_{f}^{m}\cdot \left(1-V^{F}\right)}{V^{F}}$$

**Figure 7:**
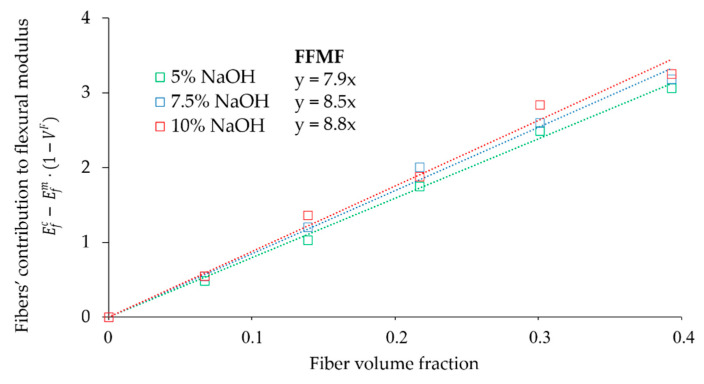
Evaluation of the Fiber Flexural Modulus Factor (FFMF).

Figure 7 presents the neat contribution of hemp core fibers treated at different NaOH concentrations against the fiber volume fractions.

**Figure 8:**
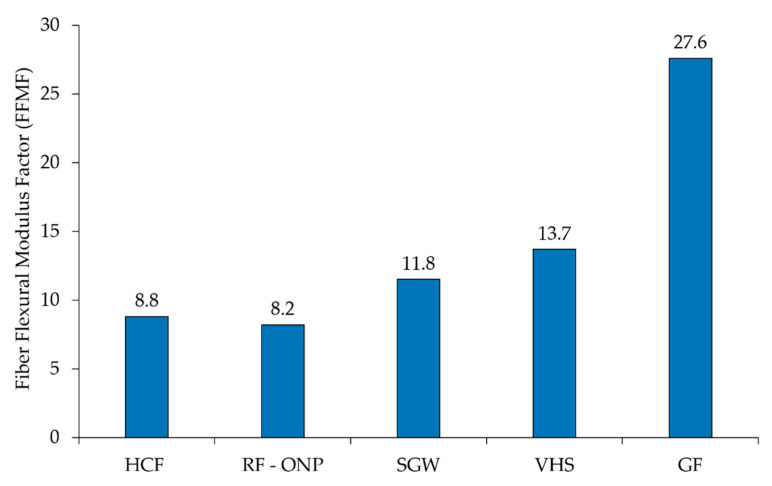
Hemp core fibers FFMF versus PP-based composites [40,63]

The FFMF values obtained were 7.9, 8.5 and 8.8 for fibers treated at 5, 7.5 and 10 wt % of NaOH, respectively. The obtained results were compared with the literature on composites using the same matrix. Figure 8 displays the FFMF of PP-based composites reinforced with hemp core fibers obtained at 10 wt % of NaOH (HCF), recycled fibers from old newspaper (RF-ONP), stone groundwood fibers from softwood (SGW), virgin hemp strands (VHS) and glass fibers (GF).

The use of GF as reinforcement resulted in substantially higher FFMF than natural fibers. GF exhibited a 3.1 times higher contribution than hemp core fibers. This roughly means that 3.1 more times the amount of hemp core fibers is needed to attain the same flexural modulus as GF composites. This can be explained by the higher intrinsic properties of GF. Despite that, glass fiber processing is high energy demanding, involves health risks and causes weathering to the equipment, apart from significant environmental drawbacks. Besides, hemp core fibers were found to be in line with the stiffening capabilities of other natural fibers. Differences are observed in their nominal values, but with little statistical significance. It is concluded that hemp core fibers show stiffening capacities comparable to other natural fibers, with the benefit of valorizing a lignocellulosic residue and avoiding the use of woody resources.

By analogy to the proposed methodology for the calculus of the FFMF, a Fiber Tensile Modulus Factor (FTMF) can be predicted by using Young’s modulus values. The ratio between the FFMF and FTMF has been proposed by some authors to compute the intrinsic flexural modulus of the reinforcements [40]. This assumption is supported by the fact that the modulus efficiency factor $\left(\eta_{e}\right)$, which is mainly influenced by the mean orientation and morphology of the fibers, does not depend upon the type of test conducted. As a result, the modulus efficiency factor calculated from the Young’s modulus or flexural modulus values should remain in the same order of magnitude. Assuming this, we obtain:

(12) $$\frac{FFMF}{FTMF}=\frac{\eta_{e}\cdot E_{f}^{F}\;}{\eta_{e}\cdot E_{t}^{F}}=\frac{E_{f}^{F}\;}{E_{t}^{F}}\;;\;E_{f}^{F}=\frac{FFMF}{FTMF}\cdot E_{t}^{F}$$

In a previous work [64], FTMF values of 12.2, 12.4 and 13.0 were recorded for PP composites charged with fibers treated at a 5, 7.5 and 10 wt % of NaOH, respectively. For the respective NaOH-treated fibers, the intrinsic Young’s moduli were, respectively, 25.7, 26.3 and 27.7 GPa. According to Equation (12), the values of the intrinsic flexural modulus of hemp core fibers are around 16.6, 18.0 and 18.3 GPa for 5, 7.5 and 10 wt % NaOH treated fibers, respectively. However, Equation (12) presents a theoretical hypothesis and needs to be confirmed by using more established models.

### 3.4. Micromechanics of the Flexural Modulus

The intrinsic flexural modulus of the fibers was computed by means of (i) the Hirsch model and (ii) the Tsai–Pagano model using Halpin–Tsai equations (TP&HT). Results are presented in Table 3.

The value of the intrinsic flexural modulus changed with the percentage of reinforcement and with the harshness of the NaOH treatment. Regarding fiber content, the intrinsic flexural modulus tended to increase with the reinforcement percentage up to 40 wt % contents, where a local maximum value was found for almost all the composites and treatments, regardless of the model used to obtain such intrinsic flexural moduli. Then, the property decreased again for 50 wt % of reinforcement. This behavior could be due to the formation of fiber bundles and aggregates as the reinforcement content increases, leading to unequal stress transferal between the non-aggregated and aggregated fibers. Finally, the efficiency of the reinforcement decreases, and the intrinsic flexural modulus of the fibers increases to keep the linear relation between the composite’s flexural modulus and fiber volume fraction. At elevated fiber contents over the 40 wt %, the intrinsic flexural modulus drops due to a reduction of fiber efficiency and the composite’s flexural modulus.

It is worth mentioning that the intrinsic flexural modulus peak of highly delignified fibers (10 wt % NaOH) was found at around 20 wt % of reinforcement. Besides, the intrinsic flexural modulus of fibers with higher Kappa numbers (5 and 7.5 wt % NaOH) was attained at larger fiber contents between 30 and 40 wt %. This corroborates the key role of lignin in promoting the dispersion of the fibers avoiding the formation of aggregates.

**Figure 9:**
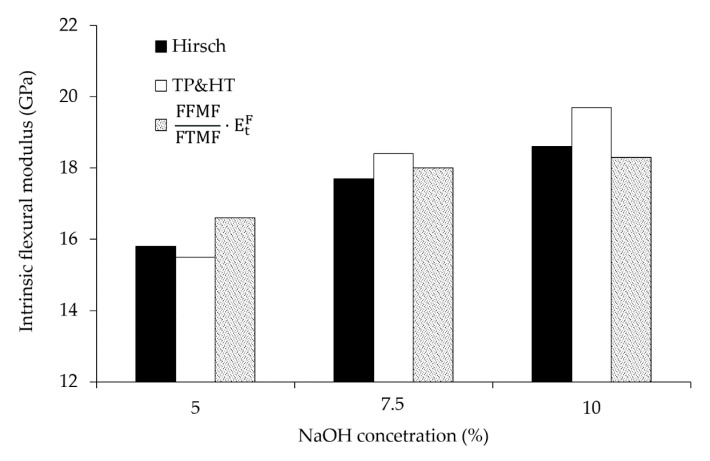
Evolution of hemp core fibers’ intrinsic flexural modulus (Hirsch; TP&HT; FFMF/FTMF) against the NaOH concentration.

The average and standard deviation of the fibers’ intrinsic flexural modulus was calculated from the values at the different reinforcement amounts. The Hirsch model accounted for 15.8 ± 0.9, 17.7 ± 1.2 and 18.6 ± 1.5 GPa at NaOH concentrations of 5, 7.5 and 10 wt %. For the same treatments, TP&HT delivered mean intrinsic flexural modulus of 15.5 ± 1.1, 18.4 ± 2.4 and 19.7 ± 3.32 GPa. Both models showed good agreement; in fact, ANOVA analysis at 95% confidence revealed no significant difference between Hirsch and TP&HT. In addition, the values obtained were comparable to the ones found by using the product of the FFMF/FTMF ratio and the fibers’ intrinsic Young’s modulus. The good agreement between methodologies suggests the usefulness and effectiveness of the Hirsch model and the FFMF/FTMF ratio to calculate the intrinsic flexural modulus. These models do not require morphological data. Similarities between models are better observed in Figure 9, where the intrinsic flexural modulus of the fibers is plotted against the NaOH concentration.

**Figure 10:**
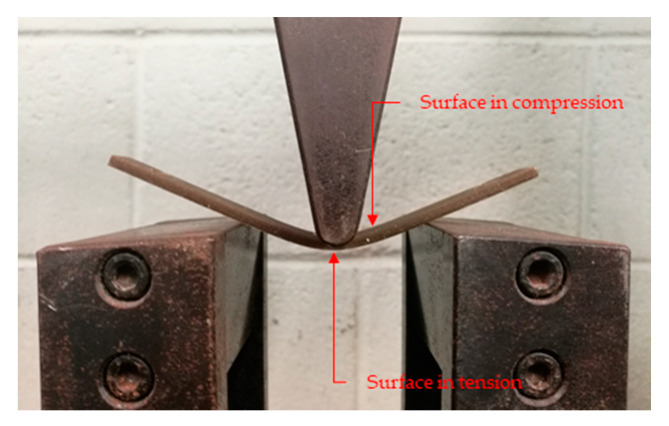
Combination of compressive and tensile loads during the three-point bending test.

Note that the fibers’ intrinsic flexural modulus was notoriously lower than the intrinsic Young’s modulus. The discrepancy arises owing to different the stress-transfer mechanism when fibers are subjected to tensile and flexural loads. During the three-point bending test, some fibers experience compression loads, whereas the rest are subjected to tensile loads (Figure 10). Owing to the highly orientated molecules chain or crystals along the fiber axis, fibers can withstand higher loads when the force is exerted parallel to their direction. However, at compression, fibers show low dependence on fiber orientation. In addition, fiber buckling and torsion take place when the fiber is submitted to compressive loads, resulting in lower performance of such fibers under compression. As a result, fibers under compressive forces will not contribute the same as the ones under tensile to the flexural modulus of the material, finally driving to a reduction of the fibers’ intrinsic flexural modulus. Thus, in semi-aligned composites, the intrinsic Young’s modulus is expected to be higher than the intrinsic flexural modulus. In the case of randomly orientated fibers, the contribution of the reinforcement to the tensile and compression modulus would equalize. Hence, as reported by Shibata, S. [70], the differences between fibers’ intrinsic flexural and Young’s modulus would decrease. It is stated then that the differences between the fibers’ flexural modulus and Young’s modulus is mainly attributed to the degree of fibers’ alignment and so the material’s anisotropy [41,71,72,73,74].

**Figure 11:**
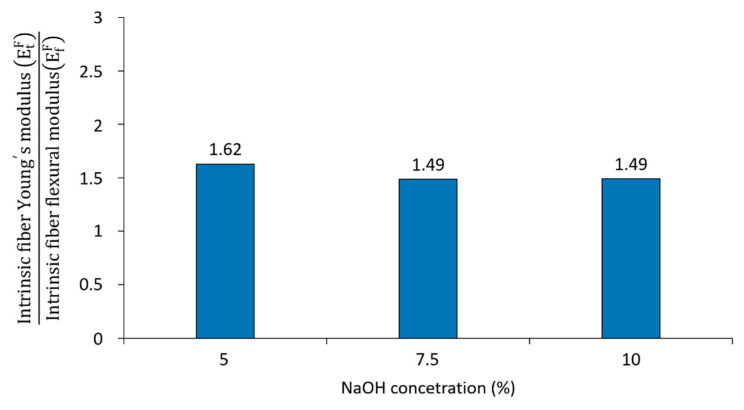
Relationship between the fiber intrinsic tensile modulus and intrinsic flexural modulus at different NaOH concentrations.

In the present work, ratios between the intrinsic Young’s and flexural moduli were found at 1.62, 1.49 and 1.49 in fibers treated at a 5, 7.5 and 10 wt % of NaOH, as observed in Figure 11. In addition, the increment of the orientation efficiency factors (Table 4) in composites is seen to stimulate the differences between the intrinsic flexural and Young’s moduli.

The implication of fibers’ morphology and orientation on the composite’s flexural modulus was evaluated via the length and orientation factor. Furthermore, the modulus efficiency factor and mean orientation angle of fibers were also obtained (Table 4).

Slight differences between the modulus, length and orientation efficiency factors were perceived between the different NaOH treatments. The modulus efficiency factors were in line with others reported in previous works, around 0.5, supporting the relevance of the results [37,60,75]. The orientation efficiency factor was studied to evaluate how the alignment of the fibers affected the fiber reinforcing effect. It has been reported that the orientation efficiency factor is 1 when fibers are completely aligned, 3/8 for planar random configuration and 1/5 for three-dimensional random orientation [76]. In the present case, values between 0.54 and 0.55 were found, exhibiting a certain degree of fiber alignment inside the matrix. This factor tended to decrease as the severity of the treatment increased, probably explained by a reduction of fiber dispersion inside the matrix at lower Kappa numbers. Besides, the high values of the length efficiency factor suggested the importance of fiber morphology on the composites’ flexural modulus of the studied composites.

From the orientation efficiency factor, a theoretical mean orientation angle of the fibers was found between 29 and 30° using a square packing approximation. The value of the mean orientation angle obtained by another route (Young’s modulus) was similar, with a defined limit angle of 54° and mean orientation angle of 29.7° [64]. However, the analysis of the tensile strength of hemp core fiber composites delivered substantially different angles [65]. In this case, the mean orientation factor was found to be $X_{1}=0.29$ which would provide a defined limit angle inside the composite of 43°, according to the relation $X_{1}=\cos^{4}\left(\alpha_{o}\right)$ established by Mittal et al. [51], and a mean orientation angle of 24.1. Anyhow, other studies have shown disagreement in the calculus of the mean orientation angle derived from the strength and modulus of composites [77,78].

Regarding the Young’s modulus micromechanical analysis [64], the modulus efficiency factors were found in the same order of magnitude as the ones obtained from the analysis of the flexural modulus. This supports the theoretical hypothesis regarding calculating the intrinsic flexural modulus from the FTMF/FFMF ratio. From the analysis of the Young’s modulus, the length efficiency factor was slightly higher than the ones obtained from the flexural modulus analysis, whereas the orientation efficiency factor was found to be below those. It is concluded that the stiffening efficiency of the reinforcement remains equal between tests. However, the Young’s modulus is more affected by the fiber morphology, whereas the flexural modulus is more influenced by the mean orientation of the fibers. This fact can be linked with the aforementioned discrepancies between the intrinsic Young’s and flexural moduli of the fibers, where the differences between both properties were found to be stimulated by the mean fiber’s orientation.

## 4. Conclusions

Hemp core residues were chemically treated at different NaOH concentrations (5, 7.5 and 10 wt %) and further defibrated via Sprout–Waldron equipment. Fibers were then incorporated into a PP matrix at different reinforcement contents ranging from 10 to 50 wt %. The flexural modulus of the composites was analyzed from a macro and micro mechanical viewpoint. At a 50 wt % of reinforcement, remarkable increments in the flexural modulus of about 239%, 250% and 257% were attained in fibers treated at 5, 7.5 and 10 wt % of NaOH, respectively. Additionally, the presence of lignin was proved to benefit the dispersion and distribution of the fibers inside the matrix. The contribution of the fibers to the flexural modulus of the composites, evaluated by a fiber flexural modulus factor, suggested the potential of hemp core residues to displace wood fibers in composites.

From the micromechanical analysis, the following remarks were highlighted: (i) The intrinsic flexural modulus was affected by the NaOH concentration treatment and fiber content in composites; (ii) the product of the FFMF/FTMF ratio and the intrinsic Young’s modulus was used to compute the fibers’ intrinsic flexural modulus, showing good agreement with more established models such as Hirsch and Tsai–Pagano; (iii) approximate ratios between the fibers’ intrinsic Young’s and flexural modulus around 1.5 were attained. Differences were attributed to differences of stress-transfer distribution between tests and fiber orientation; (iv) the efficiency factor stated the reinforcing potential of NaOH treated hemp core fibers; the length factor showed the importance of fiber morphology on the materials’ flexural modulus; and the orientation factor revealed semi-alignment of the fibers during the compounding process.

## Figures and Tables

**Table 1:** Process yield, Kappa number and morphology of hemp core fibers treated at different NaOH concentrations.

NaOH (wt %)	Process Yield (wt %)	Kappa Number	l_ww_^F^ (µm)	d^F^ (µm)	l_ww_^F^/d^F^
5	78.6	73.2 ± 0.3	655 ± 9	23.7 ± 0.4	16.0
7.5	76.4	68.1 ± 0.3	665 ± 12	24.5 ± 0.6	15.8
10	66.9	57.0 ± 0.4	684 ± 6	24.6 ± 0.6	16.1

l_ww_^F^: Mean fiber double weighted length (weighted in weight); d^F^: Mean fiber diameter; l_ww_^F^/d^F^: Aspect ratio.

**Table 2:** Flexural modulus (E_f_^c^) and maximum flexural deformation (Ɛ_f_^c^) of hemp core fiber-reinforced polypropylene composites with and without maleic anhydride polypropylene (MAPP).

			0 wt % MAPP		6 wt % MAPP	
NaOH (wt %)	Hemp Core (wt %)	V^F^	E_f_^c^ (GPa)	Ɛ_f_^c^ (%)	E_f_^c^ (GPa)	Ɛ_f_^c^ (%)
PP	-	-	1.10 ± 0.05	9.60 ± 0.12	1.10 ± 0.05	9.60 ± 0.12
5	10	0.067	1.57 ± 0.06	7.01 ± 0.13	1.51 ± 0.03	7.89 ± 0.12
	20	0.139	2.12 ± 0.09	5.52 ± 0.09	1.98 ± 0.04	6.89 ± 0.11
	30	0.217	2.67 ± 0.03	4.21 ± 0.14	2.61 ± 0.06	5.78 ± 0.11
	40	0.301	3.30 ± 0.04	2.89 ± 0.08	3.26 ± 0.04	4.90 ± 0.07
	50	0.393	3.72 ± 0.09	2.09 ± 0.09	3.73 ± 0.03	4.11 ± 0.09
7.5	10	0.067	1.59 ± 0.10	7.24 ± 0.06	1.57 ± 0.05	8.14 ± 0.12
	20	0.139	2.21 ± 0.04	5.64 ± 0.14	2.15 ± 0.06	7.26 ± 0.13
	30	0.217	2.70 ± 0.06	4.32 ± 0.15	2.87 ± 0.04	6.21 ± 0.06
	40	0.301	3.41 ± 0.04	3.35 ± 0.10	3.37 ± 0.03	5.41 ± 0.12
	50	0.393	3.90 ± 0.05	2.50 ± 0.11	3.85 ± 0.02	4.86 ± 0.06
10	10	0.067	1.58 ± 0.03	7.11 ± 0.08	1.58 ± 0.04	7.92 ± 0.11
	20	0.139	2.18 ± 0.02	5.54 ± 0.09	2.31 ± 0.04	7.12 ± 0.12
	30	0.217	2.71 ± 0.07	4.41 ± 0.13	2.74 ± 0.02	6.14 ± 0.14
	40	0.301	3.51 ± 0.02	3.41 ± 0.11	3.61 ± 0.04	5.35 ± 0.09
	50	0.393	3.93 ± 0.05	2.48 ± 0.13	3.92 ± 0.03	4.82 ± 0.11

**Table 3:** Hemp core fibers’ intrinsic flexural modulus computed by means of Hirsch and Tsai–Pagano and Halpin–Tsai (TP&HT) models at different NaOH concentrations.

NaOH (wt %)	Hemp Core (wt %)	E_f_^F^—Hirsch (GPa)	E_f_^F^—TP&HT (GPa)
5	10	14.8	15.2
	20	15.2	15.3
	30	16.6	16.7
	40	16.9	16.5
	50	15.4	14.0
7.5	10	17.0	18.7
	20	18.2	19.9
	30	19.5	21.2
	40	17.8	17.5
	50	16.2	14.9
10	10	17.4	19.2
	20	21.1	24.6
	30	18.0	18.8
	40	19.8	20.5
	50	19.6	15.4

**Table 4:** Modulus efficiency factor (η_e_), length efficiency factor (η_l_), orientation efficiency factor (η_o_), limit angle (α_o_), mean orientation angle (α).

NaOH (wt %)	η_e_	η_l_	η_o_	α_o_	α
5	0.492	0.910	0.540	53.39	29.49
7.5	0.483	0.902	0.535	53.53	29.55
10	0.479	0.902	0.531	54.10	29.83
